# Predictive Factors of Herpes Zoster HIV-Infected Patients: Another Adverse Effect of Crack Cocaine

**DOI:** 10.1371/journal.pone.0080187

**Published:** 2013-11-11

**Authors:** Mathieu Nacher, Celia Basurko, Antoine Adenis, Emilie Gaubert-Marechal, Emilie Mosnier, Sophie Edouard, Vincent Vantilcke, Sindou Sivapregassam, Benoit Tressières, André Cabié, Pierre Couppié

**Affiliations:** 1 Centre d’Investigation Clinique Epidémiologie Clinique Antilles Guyane (INSERM / DGOS CIE 802), Centre Hospitalier de Cayenne, Cayenne, French Guiana; 2 Epidemiologie Parasitoses et Mycoses Tropicales, EA 3593, Université Antilles Guyane, and COREVIH Guyane, Cayenne, French Guiana; 3 Centre Hospitalier de Cayenne, Cayenne, French Guiana; 4 Service de Dermatologie Vénéréologie, Centre Hospitalier de Cayenne, Cayenne, French Guiana; 5 Service de Médecine, Centre Hospitalier Franck de l’Ouest Guyanais, Saint-Laurent-du-Maroni, French Guiana; 6 Centre d’Investigation Clinique Epidémiologie Clinique Antilles Guyane (INSERM / DGOS CIE 802), Centre Hospitalier Universitaire de la Martinique, Fort de France, Martinique, France; 7 Service de Maladies Infectieuses et Tropicales, Centre Hospitalier Universitaire de la Martinique, Fort de France, Martinique, France; Fudan University, China

## Abstract

A retrospective cohort study was conducted on 1541 HIV-infected patients to determine variables associated with the incidence of herpes zoster. A single failure Cox model showed that herpes zoster incidence increased following the first 6 months of antiretroviral treatment adjusted hazard ratio (AHR)=5 (95%CI=2.6-9.2), P<0.001; in the >60 years age group AHR=2 (95%CI=1-4), P=0.04; in patients in the top CD8 quartile AHR=2.1 (95%CI=1.3-3.6), P<0.001; and in patients previously reported to use crack cocaine AHR=5.9, (95%CI=1.4-25), P=0.02. Herpes zoster incidence increased in patients with CD4 counts<500 per mm^3^ and gradually declined since 1992-1996, with AHR=0.3 (95%CI=0.2-0.5), P<0.001 for the 1997-2002 period and AHR=0.24 (95%CI=0.14-0.4), P<0.001 for the 2002-2008 period. Contrary to what has been described elsewhere, there was no specific effect of protease inhibitors on herpes zoster incidence. The present study is the first to suggest that crack cocaine is associated with an increased incidence of herpes zoster. The neurological or immunological effects of crack are discussed.

## Introduction

Similar to other herpes viruses, the varicella-zoster virus (VZV) causes both acute illness and lifelong latency. During the primary infection, VZV enters the cutaneous endings of sensory nerves and migrates up to the sensory nerve ganglia. The virus incorporates nucleoprotein within ganglionic cells and establishes latency which is maintained through cellular immunity [1]. With advancing age, VZV-specific cellular immunity weakens and the virus may be reactivated and migrate along the sensory nerves to reach the corresponding dermatoma. Other causes of immunodepression, such as HIV infection, may lead to the same consequences[2]. The occurrence of herpes zoster does not reliably predict the immunovirological progression of HIV infection. Several studies have suggested that incidence remained fairly stable at any stage of the HIV infection[3-5]. However, others have suggested that incidence was greater when immunosuppression worsened[3,6,7]. Several authors also reported that herpes zoster incidence transiently increased following antiretroviral therapy initiation thus representing a common form of immune reconstitution disease [8-11]. It was notably reported that protease inhibitors, perhaps because of their specific boosting effect on CD8 counts[12,13], were associated with a greater incidence of herpes zoster[8]. 

In acute VZV infection, varicella, primarily affects children in temperate areas. Thus most HIV-infected patients born in the United States and Europe have already had varicella or have been vaccinated, and therefore are not susceptible to primary infection. However, in tropical areas children often escape primary infection [14]. The objective of the present study was to determine the incidence and the predictive factors of Herpes zoster in a cohort of ethnically diverse patients followed in French Guiana.

## Methods

### HIV in French Guiana

French Guiana is the French territory where the HIV prevalence is highest. The epidemic is driven by transactional sex and perhaps by crack cocaine. Over seventy percent of the patients are foreign (from Haiti, Suriname, Brazil, Guyana, Dominican republic and other countries). The health system is the French system. All patients residing for over 3 months on the territory are eligible to receive health insurance and residence permits. The latest antiretrovirals, genotyping, viral load and other biological tests are free of charge.

### Patients

All HIV-positive patients followed in Cayenne, Kourou, and Saint Laurent du Maroni Hospitals between 1 January 1992 and 31 October 2008 were enrolled in the French Hospital Database for HIV (FHDH). Time-independent variables such as sex, nationality, mode of acquisition of HIV, and time-dependent variables such as age, CD4 cell counts, CD8 counts, HIV-1 viral loads, antiretroviral treatments, and clinical events are routinely entered by trained clinical studies technicians. Diagnoses are coded according to the 10th International Classification of Diseases. Specific details such as the location of herpes zoster could not be obtained from the database. 

### Ethical and Regulatory aspects

Patients included in the FHDH give written informed consent for the use of their records’ data. Their identity is encrypted before the data are sent to the Ministry of Health and the Institut National de la Recherche Médicale, which centralizes data from Regional coordination committees for the fight against HIV/AIDS (COREVIH) throughout France. This data collection is approved by the Commission Nationale Informatique et Libertés. 

### Study design

The study was a retrospective cohort study, using a single failure Cox proportional hazards model to evaluate the adjusted relationship between failure and explanatory variables. The failure event was the incidence of a first episode of herpes zoster. The main explanatory variables were age categorized <30, 30-39, 40/59, >60), sex, nationality, reported addictions, CD4 cell count at the time of the visit (categorized as <50, 50–199, 200–499, and >500 cells/ml), CD8 cell count categorized in 4 quartiles (<642, 642-949, 950-1362, >1362), HIV1 viral load, the presence or absence of HAART, the time since treatment started (<6 months, >6 months), and the period (1992-1996, 1997-2002, 2003-2008). The proportionality of the hazard functions was determined using Schoenfeld and scaled Schoenfeld residuals and the global proportional hazards test. Age, CD4 and CD8 cell count category at the time of HIV diagnosis, and follow-up duration were transformed into dummy variables to compare different groups with a reference group. 

In order to test the eventual influence of protease inhibitors, 2 approaches were used: first, an interaction term between the 6 month period following treatment initiation and protease inhibitors was created and added to the Cox model.; second, a nested case control study was conducted with 4 random controls sampled for each case of herpes zoster, at the time of herpes zoster. The data were analysed using STATA 12.0 (STATA Corp., College Station, Texas, USA). 

## Results

A total of 1541 subjects with 34310 observations were included, representing a total of 7159 person-years of follow-up. A total of 181 first clinical episodes of herpes zoster were recorded. There were 2 reported cases of meningeal herpes zoster, and 7 cases of disseminated herpes zoster. Table 1 shows the crude incidence rates and the adjusted hazard ratios for a first episode of herpes zoster. The overall incidence rate of herpes zoster was 2.2 per 100-person years. However, it increased during the 6 months following treatment initiation. Incidence progressively declined between 1992-1996 period, the 1997-2002 period, and the 2003-2008 period.

**Table 1:** Predictive factors of herpes zoster in HIV patients in French Guiana.

**Variable**	**Incidence rate per 100 person-years**	**Adjusted hazard ratio**	**(95% confidence interval)**	***P***
**CD4 count**				
[0-50]	2.4	3.5	(1.5-8.2)	0.004
]50-200]	3.1	4.2	(2.2-8)	<0.001
]200-350]	2.3	3	(1.6-5.9)	0.001
]350-500]	2	2.7	(1.4-5.3)	0.003
>500	0.9	Ref		
**CD8 count**				
[0-641]	1.3	Ref		
[642-949]	2.3	1.8	(1.06-3)	0.03
[950-1362]	1.8	1.2	(0.7-2.2)	0.4
>1362	2.9	2.1	(1.3-3.6)	<0.001
**Sex**				
Male	2	0.9	(0.7-1.1)	0.3
Female	2.1	Ref		
**Age group**				
<30	2	Ref		
[30-40]	2	1.2	(0.7-2)	0.5
]40-60]	2.1	1.2	(0.7-2)	0.5
>60	3.3	2	(1-4)	0.04
**Period relative to HAART initiation**				
Before HAART	2.2	Ref		
First 6 months of HAART	6.3	5	(2.6-9.2)	<0.001
More than 6 months of HAART	1.8	1.4	(0.8-2.1)	0.16
**Nucleoside & nucleotide inhibitors**				
Yes	1.7	0.58	(0.4-0.8)	0.001
No	2.6	Ref		
**Protease inhibitors**				
Yes	1.7	0.85	(0.4-1.7)	0.6
No	2.2	Ref		
**Non nucleoside inhibitors**				
Yes	1.3	0.85	(0.4-1.5)	0.6
No	2.26	Ref		
**Reported crack cocaine use**				
Yes	4.4	5.9	(1.4-25)	0.02
No	2.1	ref		
**Period**				
1992-1996	5.3	Ref		
1997-2002	2.1	0.3	(0.2-0.5)	<0.001
2002-2008	1.3	0.24	((0.14-0.4)	<0.001

After adjusting for potential confounders, such as antiretroviral treatment, CD4 counts, age and gender, this association remained significant. The interaction term testing a specific effect of protease inhibitors on the incidence of herpes zoster was not significant, thus removed from the final model. Similarly, the nested case control study did not find any specific association between protease inhibitors and herpes zoster (adjusted odds ratio=0.8 (95% CI=0.4-1.4), *P*=0.4. 

The CD4 decline was rapidly associated with an increased incidence of herpes zoster (<500 CD4 per mm3), but further immunosuppression only marginally increased the incidence of herpes zoster. Patients with CD8 counts in the 2^nd^ and 4^th^ quartiles had an increased incidence of herpes zoster. 

Patients aged 60 and over had an increased risk of herpes zoster.

Although there were few patients concerned (n=58), there was an association between crack cocaine use and herpes zoster (incidence rate 4.4 per 100 person-years when crack use was reported versus 2.2 per 100 person years when crack use was not reported. Table 2 shows the age-stratified incidences for crack users and non users. The nadir median CD4 count was not significantly different between crack users (133.5/mm^3^ (inter quartile range=241)) and non users (163/mm^3^ (SD=253)), *P*=0.56. CD8 counts at the same time were significantly lower among crack users (516/mm^3^ (inter quartile range=590)) than non users (763/mm^3^ (inter quartile range=668)), *P*=0.005. After controlling for CD4 count, crack users (failure event in this model) were less likely to be on ARV treatment than non crack users adjusted hazard ratio=0.17 (0.08-0.36), *P*<0.001. Finally, the Analysis of the proportion of crack users by period showed that there was a gradual increase in the proportion of crack users 1.1% in 1992-1996, 1.8% in 1997-2002, and 5.8 in 2002-2008 (*P*<0.001).

**Table 2:** Age-specific incidence rate of herpes zoster for crack users and non users.

**Age group**	Proportion of crack users (%)	Herpes zoster age-specific incidence rate for crack users (per 100 person years)	Herpes zoster age-specific incidence rate for non crack users (per 100 person years)
<30	1.95	3.7	2.4
[30-40]	3.9	2.4	2.4
]40-60]	2.7	7.2	3.2
>60	0.7	89 (2 patients only)	4.4

## Discussion

Although French Guiana is in the tropics where the proportion of persons infected by VSV is lower[14], the reported incidences of herpes zoster in HIV patients were of similar magnitude as the incidences described in the USA and spain [2,8,15-17]. The incidence tended to decrease since the years before HAART was available, as described elsewhere[18]. The incidence of herpes zoster transiently increased following antiretroviral therapy initiation, and was associated with increased age and increased CD8 count as described by others [8,9,11,19]. The incidence of herpes zoster seemed to increase for CD4 counts<500 per mm^3^ but more advanced immunosuppression did not seem to further increase incidence. It has been reported that protease inhibitor initiation specifically increased incidence of herpes zoster, possibly through a CD8 boosting mechanism[8]. In the present study we could not replicate the same finding. It is possible that at the time of that study, protease inhibitors were the main antiretrovirals that led to virological suppression and immune reconstitution. The important aspect was the virological suppression rather than the specific action of protease inhibitors. Here we observed that overall, nucleoside and nucleotide inhibitors, the back bone of most HAART regimens, were associated with an overall reduction of herpes zoster incidence. The exact pathophysiology of herpes zoster as an immune reconstitution event is not clear. First the immune restoration may lead to increased viral excretion followed by increased incidence of clinical herpes zoster. Another hypothesis, which should be tested, would be that herpes zoster is asymptomatically excreted in some patients and that the immune reconstitution following HAART leads to patent symptoms where viruses are excreted. 

The observation that crack cocaine was associated with an increase in the incidence of herpes zoster was a novel finding. Cocaine, a sigma1 agonist neurotropic drug, has an effect on the homeostatic balance between TH1 pro-inflammatory versus TH2 anti-inflammatory balance and skews the cytokine response towards anti-inflammatory cytokines[20]. Thus, a hypothesis would be that the immunomodulatory effects of cocaine would further increase viral excretion in patients already immunocompromised by HIV leading to herpes zoster. It has been reported that crack was associated with increased incidence of herpes [21], the present observation could also reflect a similar effect on another virus from the herpes virus family. Although the results were statistically significant, the numbers were small, therefore the findings should be replicated in larger series.
